# Construction of Various γ34.5 Deleted Fluorescent-Expressing Oncolytic herpes Simplex type 1 (oHSV) for Generation and Isolation of HSV-Based Vectors

**DOI:** 10.18869/acadpub.ibj.21.4.206

**Published:** 2017-07

**Authors:** Shahriyar Abdoli, Farzin Roohvand, Ladan Teimoori-Toolabi, Mohammad Ali Shokrgozar, Mina Bahrololoumi, Kayhan Azadmanesh

**Affiliations:** 1 Department of Virology, Pasteur Institute of Iran, Tehran, Iran; 2 Molecular Medicine Department, Biotechnology Research center, Pasteur institute of Iran, Tehran, Iran; 3 National Cell Bank of Iran, Pasteur Institute of Iran, Tehran, Iran

**Keywords:** Oncolytic viruses, Herpes simplex virus, Homologous recombination, Flow cytometry, Fluorescence microscopy

## Abstract

**Background:**

Oncolytic herpes simplex virus (oHSV)-based vectors lacking *γ34.5* gene, are considered as ideal templates to construct efficient vectors for (targeted) cancer gene therapy. Herein, we reported the construction of three single/dually-flourescence labeled and *γ34.5*-deleted, recombinant HSV-1 vectors for rapid generation and easy selection/isolation of different HSV-Based vectors.

**Methods:**

Generation of recombinant viruses was performed with conventional homologous recombination methods using green fluorescent protein (GFP) and BleCherry harboring shuttle vectors. Viruses were isolated by direct fluorescence observation and standard plaque purifying methods and confirmed by PCR and sequencing and flow cytometry. XTT and plaque assay titration were performed on Vero, U87MG, and T98 GBM cell lines.

**Results:**

We generated three recombinant viruses, HSV-GFP, HSV-GR (Green-Red), and HSV-Red. The HSV-GFP showed two log higher titer (1010 PFU) than wild type (108 PFU). In contrast, HSV-GR and HSV-Red showed one log lower titer (107 PFU) than parental HSV. Cytotoxicity analysis showed that HSV-GR and HSV-Red can lyse target tumor cells at multiplicity of infection of 10 and 1 (*P*<0.001). Moreover, HSV-GFP showed higher infection potency (98%) in comparison with HSV-GR (82%).

**Conclusion:**

Our oHSVs provide a simple and an efficient platform for construction and rapid isolation of 2^nd^ and 3^rd^ generation oHSVs by replacing the inserted dyes with transgenes and also for rapid identification via fluorescence activated cell sorting. These vectors can also be used for tracing the efficacy of therapeutic agents on target cells, imaging of neural or tumoral cells *in vitro*/*in vivo* and as oncolytic agents in cancer therapy.

## INTRODUCTION

Cancer is one of the major public health problems worldwide and is currently the second leading cause of death. This disease is expected to become the first leading cause of death in the next few years[1]. Surgery, chemotherapy, and radiotherapy are three major current approaches for cancer treatment, most of which are either insufficient to treat cancers or exhibit side effects with highly damaging potential on proliferative tissues[2]. Therefore, there is a need for novel targeted treatment strategies to improve survival rates and decrease toxicity[3]. Oncolytic virotherapy is a new approach that empowers viruses to fight cancers[3]. An oncolytic virus is a virus that preferentially infects and kills cancer cells by lysis while sparing normal cells[3]. Oncolytic viruses can carry transgenes such as immunomodulatory genes and death ligands.

Different oncolytic viruses are being studied in preclinical and clinical research, including vaccinia virus, adenovirus, herpes simplex virus (HSV), myxoma virus, reovirus, Newcastle disease virus, and vesicular stomatitis virus[4]. HSV type 1 (HSV-1) is an enveloped, double-stranded linear DNA virus whose genome is 152 kb in length consisting of unique long and short regions each flanked by inverted repeat sequences[5]. HSV-1 was identified as an attractive candidate for oncolytic virotherapy due to its several characteristics such as natural cytolytic ability, well-characterized large genome capable of inserting multiple therapeutic genes (up to 20 kb), feasibility of genetic engineering to construct recombinant vectors, broad range of hosts, as well as the availability of some approved anti-herpetic drugs[6].

Different generations of oncolytic HSV (oHSV)-based vectors have already been developed. The first generation of oHSV was constructed by inactivating γ34.5 genes[7]; there are two copies of γ34.5 in the HSV genome. Basically, host protein kinase R (PKR) phosphorylates elongation initiation factor 2 (eIF2α) in response to viral infection, resulting in the inhibition of protein synthesis. The γ34.5 reverses PKR’s action by dephosphorylating the eIF2α so that the virus can replicate and lyse normal cells[8]. Because the PKR system is inactive in a wide variety of tumor cells, γ34.5-deleted viruses can replicate and lyse tumor cells while sparing normal cells[9]. SEPREHVIR is a good example of this generation (HSV1716)[10].

The second generation of oHSV vectors harbors deletion, in addition to γ34.5 deletions, in metabolic genes such as thymidine kinase[11] or ribonucleotide reductase[12]. Products of these two genes are vital for virus replication in quiescent cells. Deletion of thymidine kinase or ribonucleotide reductase (RR) limits viral replication to dividing cells. The third generation of oHSVs is constructed by arming the first or second generations of oHSV with various effector transgenes such as immunomodulatory genes (e.g. interleukin family or granulocyte-macrophage colony-stimulating factor[13,14], tumor suppressor genes, death ligands (e.g. TRAIL), or tumor-specific promoters[15]. In this generation, tumor-killing activity is enhanced due to the effects of added transgenes. The best example of this generation is the recently FDA approved IMLYGIC™ (talimogene laherparepvec) for melanoma[16].

Homologous recombination is a basic method for the generation of recombinant HSV-based vectors[17]. This technique requires time-consuming selection processes and structural confirmation. This approach led to the development of other newer methods such as HSV-BAC[18] and HSVQuick systems[19]. Despite these novel methods, some problems still exist. It should be noted that the basis of these new approaches is the traditional homologous recombination at the first step. In addition, BAC-based methods require manipulation of BAC in bacteria, BAC extraction, transfection of BAC into mammalian cells, and packaging of recombinant viruses in host cells[18]. A common strategy to select the recombinant viruses is using a marker. Several selection markers have been used for isolation of recombinant viruses such as green fluorescent protein (GFP)[13], JRed[20], LacZ[12], and mCherry[21].

To facilitate the labor-intensive steps of selecting recombinant HSVs, we herein report the construction of three single/dually-labeled recombinant HSV-1 vectors. This information provides an opportunity to replace each γ34.5 with a distinct gene and to easily select them with fluorescent microscopy. These recombinant vectors could be used directly as a drug to kill cancer cells or as a basic platform for construction of higher oHSV generations.

## MATERIALS AND METHODS

### Cells and virus

Vero (African green monkey kidney cells), T98 GBM (human glioblastoma; NCBI- C651), U87 MG (human glioblastoma; NCBI-C531), and BHK 21 (baby hamster kidney; NCBI-C107) cells were purchased from the National Cell Bank of Iran (NCBI, Pasteur Institute of Iran, Tehran, Iran). Vero and T98 GBM cells were cultured in RPMI 1640 (Thermo Fisher Scientific, USA) supplemented with 10% FBS (Thermo Fisher Scientific, USA). U87 MG and BHK 21 cells were cultured in DMEM (Thermo Fisher Scientific, USA) supplemented with 10% FBS. The cell lines were incubated at 37°C, 5% CO_2_ and 95% humidity. HSV-1 was kindly provided as a gift by Dr. Houriyeh Soleimanjahi (Tarbiat Modares University, Iran). Virus stocks were generated from low-multiplicity infections. All cells were purchased from NCBI (Iran).

### HSV propagation and DNA extraction

Vero cells were plated into 15-cm culture dishes and incubated at 37°C and 5% CO_2_. After 24-hour incubation, the cells were infected with HSV-1 at a multiplicity of infection (MOI) of 3. On the next day, the cells were harvested after observation of total cytopathic effect. The supernatant was aliquoted, titered and stored at -70°C. The cells were collected by centrifugation at 300 ×g at 4°C for 5 minutes, resuspended in the lysis buffer (10 mM Tris-HCl, 10 mM NaCl, and 3 mM MgCl_2_) and lysed by three freeze/thaw cycles. After centrifugation at 2000 ×g at 4°C for 10 minutes, the supernatant was collected and treated with RNase A and DNAse I (both from Thermo Fisher Scientific, USA) at 37°C for two hours. Subsequently, the supernatant was treated with proteinase K (Promega, USA) and SDS (0.1%) and incubated at 56°C overnight. On the following day, viral DNA was purified by phenol/chloroform extraction and ethanol precipitation. DNA was resolved in Tris-EDTA buffer and stored at 4°C for further applications.

### Titration of progeny viruses

The plaque assay was used to determine viral titers[22]. In brief, pre-cultured Vero cells were seeded onto 6-well plates and infected with serial dilutions (up to 10 logs) of virus samples. Two hours later, virus inoculum was removed and freshly-prepared RPMI (supplemented with 2% FBS and 0.1% pooled human immune globulin) was added to the cells. The plates were incubated at 37°C for 3 to 4 days until plaques were visible. The cells were then fixed with methanol for 5 minutes and stained with Giemsa for 20 minutes to visualize the plaques. Plaques were counted, and the average number of plaques was determined. BleCherry and GFP-positive plaques were observed with a CETI inverted fluorescence microscope (Medline Scientific, UK).

### Cell viability and cytotoxicity assay

The cytotoxicity of recombinant HSV vectors was determined on U87MG and Vero cell lines using the Cell Proliferation Assay Kit II (XTT; Sigma, Germany). Briefly, 2.5×10^4^ cells per well were seeded onto a 96-wells plate and incubated at 37°C overnight. The next day, cells were infected with different MOIs of wild-type HSV-1, HSV-GR (Green-Red), and HSV-GFP (MOI 3, 1, 0.1, 0.01, and 0.001). After 72 hours, the medium was discarded, and 50 µl of fresh RPMI was added to each well. The XTT reagent (45 µl/well) was added to the wells, and the plates were incubated at 37°C for additional three hours. Absorbance was subsequently measured using Synergy 4 Multi-Mode Reader (Biotek, USA) at 450 nm (with 630 nm as a reference).

### Recombination plasmid constructions

#### Generation of GFP shuttle vector

**Fig. 1:**
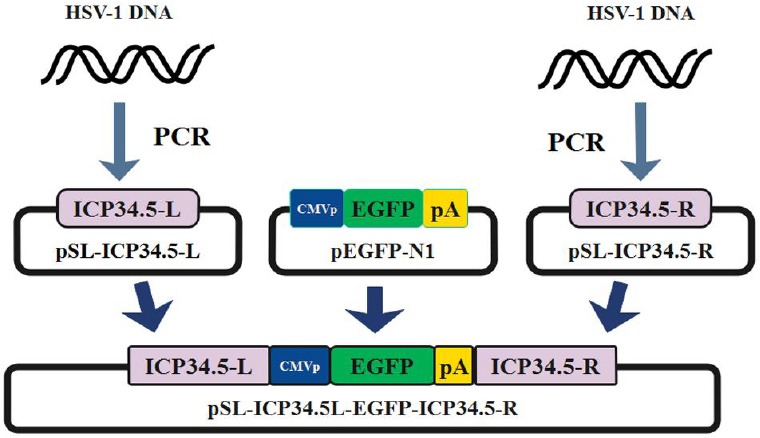
Schematic diagram of green fluorescent protein (GFP) shuttle vector construction. ICP34.5-L (left) and ICP34.5-R (right) homologous arms were amplified using specific primers (Table 1) to perform homologous recombination. The GFP cassette was subcloned from pEGFP-N1, as a reporter gene donor. Finally, the pSL-ICP34.5L-EGFP-ICP34.5R vector was generated with GFP reporter gene for generation of the recombinant virus.

The homologous fragments and reporter gene were inserted into pSL1180 (Amersham Biosciences, USA) plasmid to generate a shuttle vector. Figure 1 represents the cloning strategy used in this study. In brief, ICP34.5-R and ICP34.5-L fragments were constructed by PCR using specific primers (GH1 and GH2) (Table 1). After amplification, the fragments were cloned into the pTG19T/A (Vivantis, USA) vector and sequenced. ICP34.5L was digested with *Hin*dIII, treated with fast alkaline phosphatase (Thermoscientific, USA) and cloned into pSL1180 to generate pSL-ICP34.5L. Subsequently, ICP34.5R was subcloned into pSL-ICP34.5-L at *Bam*HI and *Bgl*II sites to make pSl-ICP34.5L-ICP34.5R. Furthermore, GFP expression cassette-CMV promoter-EGFP-BGH poly A signal was subcloned from pEGFP- N1 (Clontech Laboratories, USA) into pSL-ICP34.5L-ICP34.5R using *Bgl*II and *Afl*II (Fig. 1). Finally, pSL- ICP34.5L-CMV-EGFP-pA-ICP34.5R was confirmed by restriction analysis and used to insert GFP into the γ34.5 site.

**Table 1:** Primer sets for generating homologous fragments and recombination analysis.

Primer	Sequence
HSV-GFP construction	
GH1-Fw	AGAAGATCTGAGTAGTGCTTGCCTGTCTAACTCG
GH1-Re	AAAGGATCCCGACCTGATTAAGTTTTGCAGTAGCG
GH2-Fw	TAGAAGCTTGAGGTGCAAATGCGACCAGACCG
GH2-Re	TGAAAGCTTAGTGGCTCCACGTTCCCGAGTC
HSV-GR and HSV-Red construction	
Homo-F1 Fw	ATGAATTCCCTAGATGCGTGTGAGTAAG
Homo-F1 Re	TAGGTACCACGACGATGACGACGACTG
Homo-F2 Fw	AGGATCCCGCAGCAGCAGCGAACAAGAAG
Homo-F2 Re	TACTGCAGGTGGATGAGGAACAGGAGTTG
Recombination analysis	
ICP34.5 Fw	TGAATTCGGCACGCTCTCTGTCTCCA
ICP34.5 Re	CAAGCTTCGGCTCCTGCCATCGTCTCTC
HSV-Control	AGGATCCAGCGAGTTAGACAGGCAAG

The ICP34.5 primers were used for the first recombination and construction of HSV-GFP. HomoF primers were used for the second recombination and construction of HSV-GR and HSV-Red.

#### Generation of BleCherry-containing shuttle vector

**Fig. 2:**
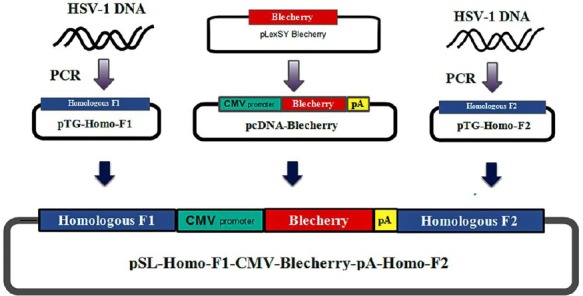
Schematic diagram of BleCherry shuttle vector construction. Homo-F1 and Homo-F2 homologous arms were amplified by PCR using HomoF-1 and Homo-F2 primer sets. The BleCherry reporter gene was subcloned from pLexSY into pcDNA3.1+ to construct the reporter cassette. Three fragments were subcloned sequentially into pSL1180. Finally, pSL-Homo-F1-CMV-BleCherry-pA-Homo-F2 was generated with the BleCherry reporter gene.

The BleCherry reporter system was used for the second homologous recombination. The schematic pattern is shown in Figure 2. Briefly, homologous arms (we named Homo-F1 and Homo-F2) were generated using PCR with specific primers (Homo-F1 and Homo-F2) (Table 1). Fragments obtained from PCR amplification were cloned into the pTG19T/A vector and sequenced. Then Homo-F1 was digested by *Hind*III and *Sac*I and sub-cloned into pSL1180 to generate pSL-Homo-F1. Homo-F2 was digested by *Eco*RI and *Bgl*II, and cloned into pSL-Homo-F1 to generate pSL-Homo-F1-Homo-F2. The BleCherry Reporter cassette was constructed by sub-cloning a BleCherry sequence from the pLexSY BleCherry vector (Jena Bioscience, Germany) into pcDNA3.1+ (Invitrogen, USA) using *Bam*HI and *Spe*I. Finally, CMV-BleCherry-pA was sub-cloned into pSL-Homo-F1-Homo-F2 at *Mlu*I and *Eco*RV sites to generate pSL-Homo-F1-CMV-BleCherry-pA-Homo-F2 shuttle plasmid (Fig. 2). The final construct was confirmed by restriction analysis and used to insert BleCherry into another γ34.5 site.

### Generation of HSV-GFP, HSV-GR, and HSV-Red

The day before transfection, BHK21 cells were seeded onto 6-well plates and incubated at 37°C and 5% CO_2_. Afterwards, pSL-ICP34.5L-CMV-GFP-pA-ICP34.5R or pSL-Homo-F1-CMV-BleCherry-pA-Homo-F2 was transfected to BHK cells using lipofectamine 2000 (Invitrogen, USA) according to the manufacturer’s instructions. Transfected cells were incubated at 37°C overnight. The next day, cells were observed with an inverted fluorescent microscope to investigate fluorescent dye expression. Subsequently, transfected cells were infected with wild-type HSV-1, as mentioned above. Briefly, medium was discarded, and the cells were washed with PBS. HSV-1 virus was added to the cells at MOI of 1. After two hours, the infection media were removed and fresh DMEM (supplemented with 2% inactivated FBS) was added to the cells. Forty hours later, cells and supernatants were collected and stored at -70°C for further usage.

### Isolation and purification of recombinant virus

The plaque purification method was applied to isolate recombinant viruses. Vero cells were cultured on 6-cm dishes. Viruses from the recombination step were diluted to 10^-5^. Confluent monolayer Vero cells were infected with different dilutions of viruses and overlaid by RPMI containing 2% inactivated FBS and 1.5% methyl cellulose. After 48 hours, plaque formation was analyzed by an inverted fluorescent microscope, and positive plaques were marked. Next, positive plaques were isolated, and viruses were released by three freeze/thaw cycles. The infection steps were repeated four more times. Finally, the isolated recombinant viruses were cultured, titered and stored at -70°C for further applications.

### Flow cytometry assay

Infection efficiency of recombinant viruses was analyzed by flow cytometry. Vero, U87MG, and T98 GBM cells were infected with different MOIs of recombinant viruses. After 22 hours, the infected cells were trypsinized, collected and washed with PBS. Finally, the cells were resuspended and analyzed with CyFlow (Partec, Germany).

### Statistical analysis

Statistical analysis was carried out using graph pad prism 6 software. Multiple comparisons were performed with one-way ANOVA. *P* values less than 0.05 were considered to be statistically significant. Results were expressed as mean±SD.

## RESULTS

### Generation of recombinant HVS-1-based vectors

**Fig. 3:**
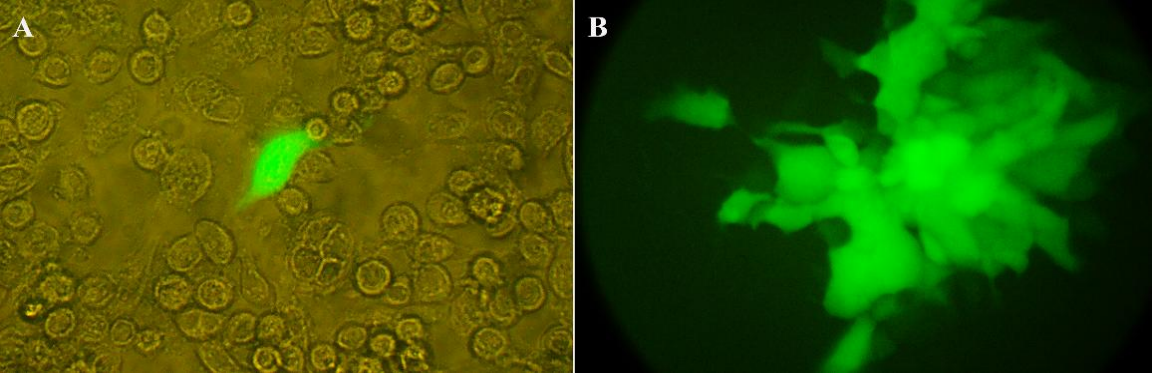
Analysis of green fluorescent protein (GFP)-positive infected cells. The green stain shows GFP expression by viral vector. (A) a single cell expressing GFP and (B) a single plaque showing GFP expression.

**Fig. 4:**
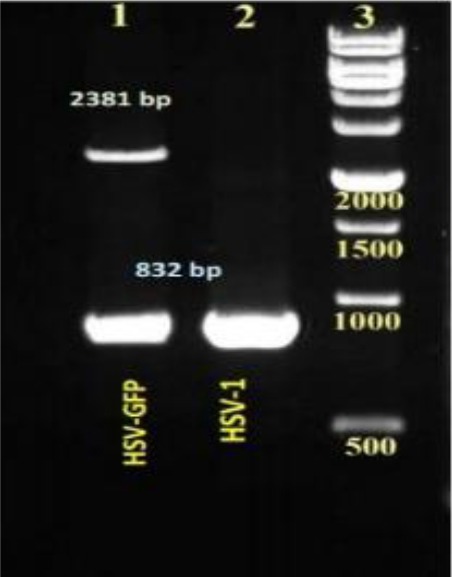
PCR analysis of herpes simplex virus (HSV)-green fluorescent protein (GFP) recombination. PCR was performed using DNA isolated from wild type and HSV-GFP by specific primers listed in Table 1. Lane1, a PCR product of recombinant virus showing two distinct bands of 832 and 2381 bp; Lane 2, wild-type virus indicating only an intact γ34.5 band (823 bp) lane 3, 1 kb DNA ladder.

**Fig.5:**
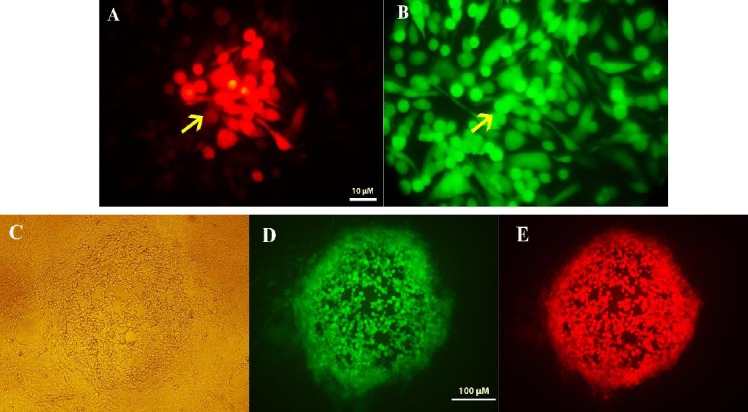
Fluorescent analysis of recombinant herpes simplex virus (HSV)-Green-Red (GR) and single plaque isolation. HSV-GR is a double-fluorescent positive virus. (A) Recombinant viruses with HSV-BleCherry-positive infected cells. Arrows indicate mCherry signals; (B) previous cells with green filter; (C, D, and E), a single plaque isolate of HSV-GR.

**Fig. 6:**
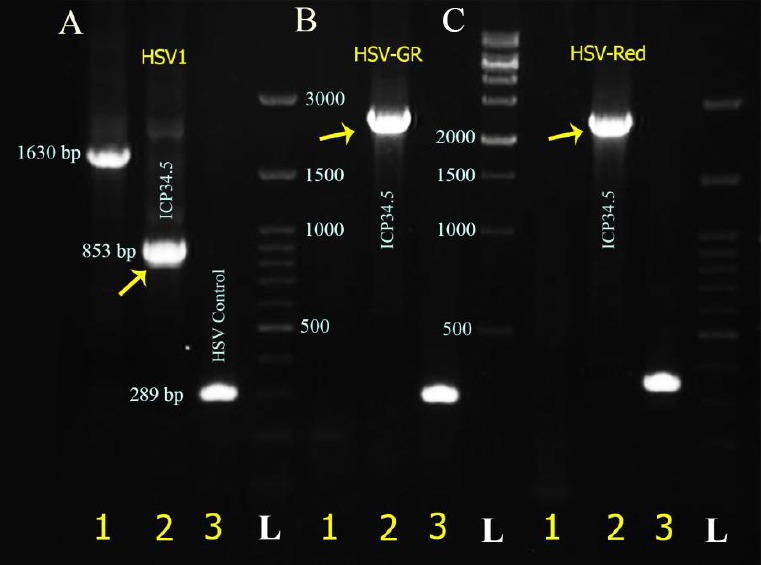
PCR analysis of recombinant herpes simplex virus (HSV)-Green-Red (GR) viruses. HSV DNA was extracted from infected cells. PCR was performed using specific primers listed in Table 1. Insertion of GFP or BleCherry increases the size of PCR products at γ34.5 loci. Lane 1, ICP34.5 and Homo-F1 set primers; lane 2, ICP34.5 forward-reverse; lane 3, HSV control. (A) HSV-1, (B) HSV-Red, and (C) HSV-GR. arrows show the band shifts at ICP34.5 cassettes. L, 1 Kb DNA ladder.

In the first recombination step, the GFP-expressing cassette was inserted into ICP34.5 locations at the HSV genome. Recombinant viruses were then isolated using the plaque purification procedure (Fig. 3). To verify recombination, viral DNA was extracted (as mentioned above) from the recombinant viruses, and PCR was performed using the HSV control and ICP34.5-R primers (Table 1). After electrophoresis, GFP-positive recombinant viruses showed two distinct bands, 832 bp and 2381 bp (Fig. 4). Wild-type HSV-I showed only the 832-bp band. This pattern indicates the inactivation of one copy of ICP34.5 genes. A similar recombination in HSV-1 was performed for construction of HSV-Red. The same procedure was applied to generate HSV-GR using HSV-GFP as the parental vector (Fig. 5A and 5B). Recombination of HSV-Red and HSV-GR were confirmed by PCR electrophoresis using three pairs of primers (Table 1). As shown in Figure 6, band shifts were observed in lane 2 for two recombinant viruses. The data shows that deletion and insertion were performed at the both ICP34.5 sites. PCR products were then sequenced to confirm recombination.

### Effects of γ34.5 deletion on the titer of progeny HSV-GFP, HSV-Red, and HSV-GR recombinant viruses

**Fig. 7:**
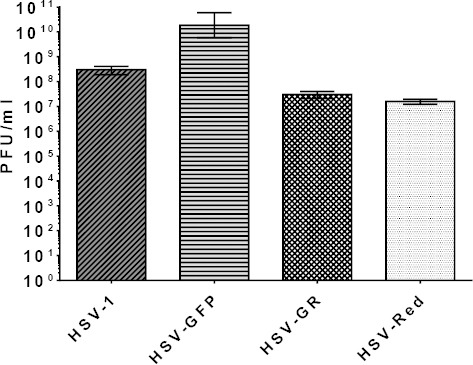
Titration of wild-type and recombinant viruses. Vero cells seeding onto 6-well plates were infected by serial dilution of wild-type and recombinant HSVs. After three days, the cells were fixed by methanol and stained with Giemsa.

Recombinant viruses were titered to determine growth properties in cell culture. Cultured Vero cells were infected with HSV-1, HSV-GFP, HSV-Red, and HSV-GR at MOI of 3. Progeny viruses were harvested and titered with the standard plaque assay on Vero cells. The mean titer of HSV-I was 2×10^8^ PFU/ml. HSV-GFP exhibited two logs higher titer than HSV-1 (above 10^10^). HSV-Red yielded 3×10^7^, and the titer of HSV-GR was 1.7×10^7^ (Fig. 7). As shown in Figure 7, HSV-Red and HSV-GR displayed lower titers than wild-type viruses.

### Cytotoxicity of recombinant viruses *in vitro*

**Fig. 8:**
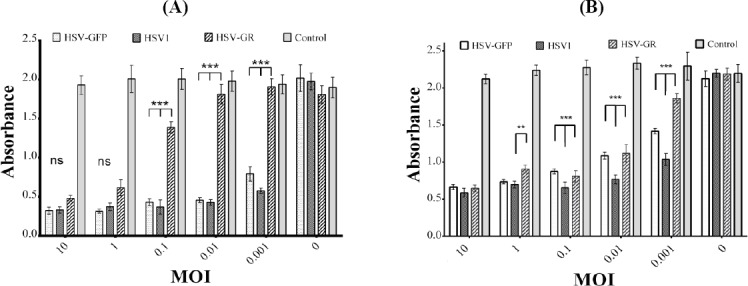
Cell viability analysis using XTT. U87MG and Vero cells were seeded onto 98-well plates. The next day, the cells were infected by HSV-1, HSV-GFP, and HSV-GR at five MOIs (10, 1, 0.1, 0.01, and 0.001). After three days, cell viability was measured using XTT reagent. At MOIs 10 and 1, all viruses showed high cytotoxicity and induced cell death. HSV-GR showed significantly lower cytotoxicity at MOI 0.1 and lower at both U87 and Vero cell lines (*P*<0.001). HSV-GFP revealed the same effect as HSV-1 on U87 cells but showed lower cytotoxicity in Vero cells at MOI 0.1 and lower, like HSV-GR (*P*<0.001). ns, not significant; **significant and ***very significant. (A) U87MG; (B) Vero.

To determine the effects of recombinant herpes virus vectors *in vitro*, Vero and U87MG cells were infected with five different MOIs of recombinants and wild-type HSV-1. All viruses showed efficient cytotoxicity at MOIs of 10 and 1, as compared to uninfected cells (*P*<0.001; Fig. 8). No significant difference was found in the cytopathic effect between wild-type and recombinant viruses at MOIs of 1 and 10 in both cells. In contrast, there was significant lower toxicity in cells infected by HSV- GR compared to HSV-1 and HSV-GFP at MOI of 0.1, 0.01, and 0.001 (*P*<0.001; Fig. 8).

Furthermore, our results showed that Vero cells are more susceptible to recombinant HSV infection than U87MG (Fig. 8).

### Infection potency of recombinant viruses

**Fig. 9:**
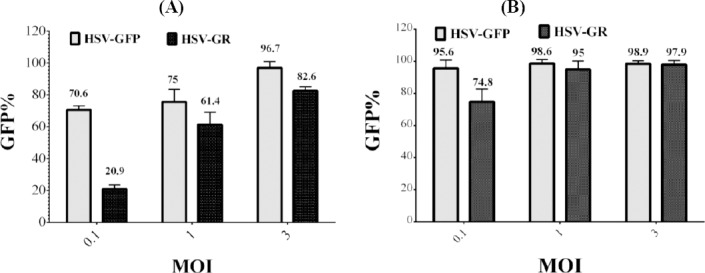
Flow cytometry analysis of HSV-GFP and HSV-GR infection. U87 MG and T98 GBM were seeded on 12-well plates and infected with different MOIs of HSV-GFP and HSV-GR (0.1, 1, and 3). After 24 hours, infection efficiency was analyzed with flow cytometry using GFP. HSV-GFP efficiently infects and replicates within and lyses both U87MG and T98 GBM cell lines at different viral louds. On the other hand, HSV-GR is less effective at low MOI in U87MG cells but has a similar infection pattern in T98 GBM cells. HSV-GR was attenuated due to the inactivation of both copies of γ34.5 genes. (A) U87 MG; (B) T98 GBM.

To measure the infection potency of recombinant viruses, U87 MG and T98 GBM cells were cultured and infected with different MOIs of recombinant HSV-GFP and HSV-GR. As illustrated in Figure 9, HSV-GFP exhibited an increase in infection potency, when compared to HSV-GR. HSV-GFP could infect more than 95% of both cell lines at MOI of 3, as compared to 82% for HSV-GR. At MOI of 0.1, HSV-GFP infected 70.6% of U87 MG cells in comparison with 20.9% of HSV-GR. Both HSV-GFP and HSV-GR showed similar infection patterns in T98 GBM at MOIs of 1 and 3. However, the infection rate of HSV-GFP and HSV-GR were 95% and 74% at MOI under 0.1, respectively, on T98 GBM (Fig. 9).

## DISCUSSION

It has been demonstrated that oncolytic HSV is a promising tool for treating various types of cancers[23,24]. Therefore, HSV-based vectors provide an ideal platform to construct novel anticancer agents. In most of the current oHSV vectors, both copies of γ34.5 genes are deleted or selectively expressed under the control of specific promoters for restricting viral toxicity to tumor cells[25]. This kind of oHSV vectors could be applied alone[23] or in combination with conventional tumor therapies[24]. Further improvement of oHSV virotherapy could be achieved by adding new potential anti-tumor agents at deleted γ34.5 gene sites.

In the present study, a double fluorescent expressing oHSV, named HSV-GR, was constructed through the deletion of both γ34.5 copies and insertion of GFP and BleCherry coding sequence into deletion sites. Moreover, a single-deleted γ34.5 gene, HSV-GFP, and a double-deleted γ34.5 gene with single fluorescent, HSV-Red, were generated. These vectors could be isolated easily by fluorescent microscopy or fluorescence activated cell sorting for rapid isolation or enrichment of recombinant viruses[25]. This property is more evident with HSV-GR. New recombinant viruses could be easily detected and separated from other viruses by the selection of one specific color (e.g. green) and loss of another color (e.g. red), respectively. These vectors were constructed by conventional homologous recombination approach[17]. However, up to our best knowledge, this is the first report of replacing both copies of γ34.5 with two distinct fluorescent dyes. In addition, we generated a single-deleted γ34.5 virus, HSV-GFP, which has not been reported so far and has shown that it gives higher titers than common dually deleted γ34.5 strains, as well as the original wild-type strain. Deletion of γ34.5 has been shown to attenuate HSV-1-based viruses[26].

Different domains of ICP34.5 had been revealed to have various functions. Previous studies have shown that the carboxyl domain of γ34.5 is essential for interaction with protein phosphatase 1 and subsequent reactivation of eIF2α; deletion of this part leads to attenuation in HSV vectors[8]. Using two different shuttle plasmids to introduce two different reporter genes led HSV-GR virus to have different deletions at each copy of γ34.5 genes (Fig. 6). In one copy, the amino-terminal end containing the ATG start codon was replaced by GFP, while carboxyl terminal of another γ34.5 copy was replaced with BleCherry (Fig. 5). In HSV-Red, both copies of γ34.5 genes were inactivated via deletion of a 325-bp fragment from carboxyl terminus and insertion of BleCherry-coding sequence in the deleted site of γ34.5. In HSV-GFP, one copy of γ34.5 genes was inactivated through insertion of a GFP-expressing cassette (Fig. 3), leaving the other copy intact.

Deleting ICP34.5 has often been shown to decrease the titer of recombinant viruses. Nakamura *et al*.[27] have demonstrated that R3616 (in which both copies of γ34.5 genes were deleted) yielded a 10-fold lower titer than strain F at HT29 cell line. However, Smith *et al*.[28] showed that both strains F and R3616 yielded around 10^7^ PFU/ml in Vero cell. Our parental clinical isolate yields one log higher titer (Fig. 7) in comparison with HSV strain F (2×10^8^ PFU/ml). This result supports the idea that fresh clinical isolates are more effective than high passage approved HSV-17 and HSV-F or HSV-KOS in viral vector production[13]. HSV-GR and HSV-Red displayed similar titers at Vero cell line (10-fold lower than the parental strain). On the other hand, Kanai *et al*.[26] revealed that the HSV1716 titer was lower than 10^7^ on Vero cell, while our HSV-GR yielded over 10^7^ PFU in the same cell line. A previous study has shown that clinical isolates are more effective at killing cancer cells than standard strains[13]. Much to our surprise, HSV-GFP virus exhibited 3 logs higher titer than strain F and 2 logs higher than its parental strain (Fig. 7). In a comparative study, Tanaka *et al*.[29] constructed a GFP expressing HSV without deletion of any viral gene, but their virus yielded 10^7^ PFU/ml. The reason of this increase is still under investigation, though there are hypotheses that it may enhance the packaging efficiency of the viral genome. These results also indicated that deletion of carboxyl domain is enough for attenuation of herpes viruses.

Deletion of γ34.5 gene has been demonstrated to decrease replication competency of recombinant virus in comparison with parental virus[30,31]. In our study, attenuation of the new viruses were tested by their cytotoxicity potential in U87 and Vero cell lines. Our data clearly demonstrated that deletion of both copies of γ34.5 genes significantly attenuated the recombinant viruses HSV-GR and HSV-RED, while inactivation of just one copy of γ34.5 genes led to no (T98 GBM) or slight (Vero) effects on viral proliferation and killing potency (Fig. 8). Furthermore, it has been reported that some cell lines are more sensitive to HSV infection than other cell lines[26]. In our experiment, the difference of cytotoxic effect of dually replaced γ34.5 strains was more obvious in lower MOIs in U87 cell line than T98 GBM (Fig. 9). We had also tested our recombinant viruses on peripheral blood mononuclear cells (as normal cells) in another experiment. After infection with low MOI, we observed that viral replication was limited and the majority of the cells were remained alive and uninfected (data not shown).

Previous studies have shown that viral vectors are ideal tools for tracking and imaging of tumor cells[32,33]. These vectors are applicable for imaging and tracking of both tumor loads and therapeutic fate *in vitro* and *in vivo*[32,34] Sizemore *et al*.[35] have reported that combination of viral vector technology with the imaging technique enhances knowledge gained from neuroanatomical studies; to this end, fluorescent-expressing vectors are essential. Our vectors have potential to be used as tools for *in vitro* and *in vivo* imaging of infected cells, especially for neuronal system. Our HSV-GR has the ability to rapidly become an armed vector by replacing one fluorescent dye with a therapeutic gene. The remaining fluorescent dye could be applied for imaging or tracking of new viral vector efficacy on target cells. Such an approach has been reported in other studies showing the capability of tracking mesenchymal stem cells carrying mCherry-expressing oHSV[30,36]. As Rochette *et al*.[37] has mentioned, mCherry signal, which is the fluorescent part of BleCherry, is above the auto-fluorescence background with limited photo bleaching for detection of cell bodies. A similar report supports replacing the GFP with the new transgene and rely on BleCherry for imaging[32]. However, many researchers have reported using GFP-labeled HSV for *in vivo* tracking[18,29]. HSV-GR can help researchers to choose more convenient dye for tracking. Due to the activity of one γ34.5, our HSV-GFP behaves more like a wild-type strain; therefore, it can be used for imaging and tracking the virus-infected cells in non-oncolytic HSV-1 studies as well.

HSV-GFP showed greater growth characteristics than the wild-type HSV-1 in different cell lines. This deletion did not alter virus killing properties but changed viral titers (Fig. 7). It seems that one copy of active γ34.5 is enough for normal replication and proliferation of HSV-1. Interestingly, HSV-GFP resulted in a higher titer than HSV-1 at the same propagation conditions as described above. HSV-GFP can be used as a reporter agent in screening anti-viral components or viral therapy procedures[38,39]. Using such a virus, the effect of anti-viral reagents can be easily measured using flow cytometry, which is a simple, rapid, sensitive, quantitative, and cost-effective approach[38,40]. Analysis of other reporter products, such as luciferase or β-galactosidase, requires expensive substrate reagents, labor working procedures, and expensive laboratory instruments.

**Fig. 10:**
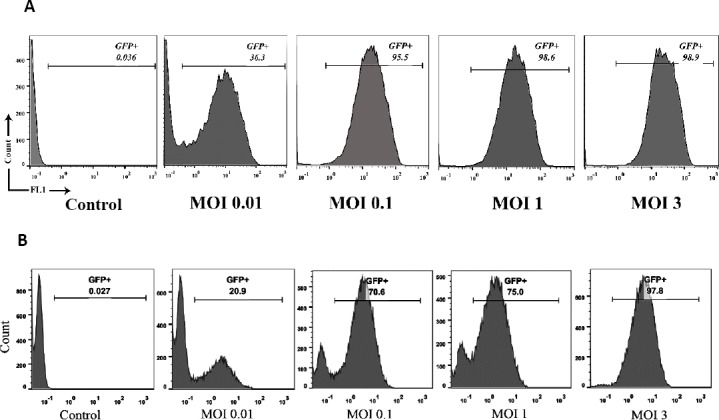
Infection efficiency of HSV-GFP on U87MG and T98 GBM cells. Plated T98 GBM and U87MG cells were infected by HSV-GFP at MOIs 3, 1, 0.1, and 0.01. After 24 hours, viral infection was analyzed by flow cytometry. At MOIs 1 and lower, T98 GBM is more sensitive to HSV-GFP infection than U87MG. (A) T98 GBM cells; (B) U87MG cells.

Investigations have indicated that some tumor cell lines are resistant to oncolytic virotherapies[32,41]. Wang *et al*.[41] has demonstrated that neuroblastoma tumor cells vary in their sensitivity to HSV infection. In our study, results from flow cytometry showed that glioblastoma cell lines exhibit the same infection pattern (Fig. 10). As shown in the Figure, T98 GBM is more susceptible to HSV infection than U87MG at lower MOIs. A few studies have shown that the therapeutic benefits of oHSVs alone appear to be limited in cancer patients[32,42,43]. In this case, armed oHSVs are more effective in killing of such tumor cells[32], which is easily attainable by our HSV-GR.

In conclusion, the three HSV vectors, constructed in this study using homologous recombination method, could be applied as oncolytic agents alone or in combination with conventional therapies. Furthermore, our vectors are suitable for imaging of neural or tumor cells both *in vitro* and *in vivo*. In addition, these vectors can open a new window for tracing the efficacy of therapeutic agents on target cells. Lastly, these vectors could be used as a platform for constructing next generations of oHSV vectors.
